# Factors Associated with Treatment Failure in Patients with Acute Exacerbation of COPD Admitted to the Emergency Department Observation Unit

**DOI:** 10.1155/2020/8261375

**Published:** 2020-06-29

**Authors:** Wasuntaraporn Pethyabarn, Sareeman Chewae, Ar-aishah Dadeh

**Affiliations:** Department of Emergency Medicine, Songklanagarind Hospital, Faculty of Medicine, Prince of Songkla University, Hat Yai, Thailand

## Abstract

**Objective:**

We aimed to identify factors associated with treatment failure in patients with acute exacerbation of COPD (AECOPD) admitted to the emergency department observation unit (EDOU).

**Methods:**

A retrospective cohort study was conducted between January 1, 2013, and October 31, 2019. The electronic medical records were reviewed of patients with AECOPD admitted to the EDOU. The patients were divided into treatment failure and treatment success groups. Treatment failure was defined as prolonged stay at the EDOU (>48 h) or COPD-related ED revisit (within 72 h) or readmission within 1 month. The two groups were compared and analyzed using univariable and multivariable analyses by logistic regression.

**Results:**

Of the 220 patients enrolled, 82 (37.3%) developed treatment failure. Factors associated with treatment failure included arrhythmias (odds ratio [OR] 3.8, 95% confidence interval [CI] 1.04–13.9), diabetic mellitus (OR 2.32, 95% CI 1.09–4.95), long-term oxygen therapy (OR 2.89, 95% CI 1.08–7.72), short-acting beta-agonist use (OR 6.06, 95% CI 1.98–18.62), pneumonia findings on chest X-ray (OR 3.24, 95% CI 1.06–9.95), and ED length of stay less than 4 h (OR 2, 95% CI 1.08–3.73).

**Conclusion:**

Arrhythmias, diabetic mellitus, long-term oxygen therapy, short-acting beta-agonist use, pneumonia findings on chest X-ray, and ED length of stay <4 h were the significant factors associated with treatment failure of AECOPD to which physicians at the ED should pay special attention before the admission of patients to the EDOU.

## 1. Introduction

Acute exacerbations of chronic obstructive pulmonary disease (AECOPD) are undesirable events in the course of the disease for most COPD patients. Previous studies showed a significant burden of exacerbations on a patient's health-related quality of life, disease progression, mortality, and costs [1–3]. However, each patient may vary in severity and outcome of an exacerbation. The outcomes can be complete recovery in a short period of time, hospitalization, or death [4].

The disposition of AECOPD patients is a challenge due to the cause, severity, impact, and treatment. Furthermore, the time course of exacerbations varies from patient to patient and there are no standards that can be applied to the timing and nature of discharge [5]. The decision to admit or discharge is often taken on the basis of clinical criteria identified in the emergency department (ED) or established by the treating physician [6]. The appropriate disposition decreases mortality and results in cost-effectiveness [5]. However, inappropriate discharge of patients leads to poor outcomes in the following weeks [7].

The emergency department observation unit (EDOU) is a designated area near the ED that is designed to accommodate patients who require a short period of observation or therapy, yet do not require inpatient admission to a ward. It is useful in decreasing the number of admissions and reducing health care costs for management by treatment, observation, and discharge of patients within 48 h [8]. A previous study stated that the availability of an EDOU can decrease hospital AECOPD admissions without affecting the number of patients discharged directly from the ED [8].

However, EDOU admissions may cause treatment failure if the patients are inappropriately selected [9]. Physicians can prevent treatment failure in the EDOU by improving the selection criteria for EDOU admission. Therefore, identifying the factors associated with treatment failure is necessary.

A previous study evaluated the factors affecting unpredictable adverse events after admission to the EDOU. The authors of that study found that the appropriateness of admission to an EDOU consisted of elderly patients, cirrhotic patients, cardiovascular patients, and an ED qSOFA score >2. These factors were associated with unpredictable adverse events in the EDOU [10]. However, that study did not focus on AECOPD patients.

Previous studies evaluated factors associated with adverse outcomes in hospitalized patients with AECOPD [4, 11, 12]. Some studies evaluated the factors associated with hospitalization of AECOPD patients in the ED [13]. However, no previous study evaluated the factors associated with treatment failure in patients with AECOPD who were admitted to the EDOU. Therefore, this study aimed to identify the factors associated with treatment failure in patients with AECOPD who were admitted to the EDOU. The identified factors will be used to develop the criteria to appropriately select AECOPD patients who need a short period of observation and treatment.

## 2. Materials and Methods

### 2.1. Study Design and Setting

A retrospective cohort study was conducted at the EDOU of Songklanagarind Hospital which is an academic, tertiary care hospital on the campus of the Prince of Songkla University in southern Thailand. The EDOU is a 15-bed short-stay unit located in the hospital having an ED volume of over 45,000 patient visits per year. The EDOU is staffed by two attending physicians (one emergency medicine staff physician and one emergency medicine trainee) for 8 h per shift, 3 shifts per day, and has round-the-clock nurse practitioner coverage. The EDOU admission criteria are age over 15 years, discharge prediction within 24 h, nonsevere illness, stabilized hemodynamics, and no requirement for intensive monitoring [10].

### 2.2. Selection of Participants

The data were collected from the computerized hospital information system between January 1, 2013, and October 31, 2019. The hospital identification numbers of the patients who visited the ED during the study period and diagnosed as J44.0, J44.1, J44.8, and J44.9 according to the ICD-10 system were compiled. One reviewer then reviewed the data from the electronic medical records.

The inclusion criteria were (1) age >40 years, (2) patients diagnosed as AECOPD, and (3) patients admitted to EDOU by the emergency physician. The exclusion criteria were (1) patients attended by other specialties, (2) patients who had incomplete essential information on their electronic medical records, and (3) patients admitted by other diagnoses but developed AECOPD in the EDOU. The included patients had to fulfill the COPD criteria set by the Global Initiative for Chronic Obstructive Lung Disease (GOLD) that included a postbronchodilator FEV1/forced vital capacity of <0.7 that was performed in the stable phase prior to EDOU admission [5]. The standard orders for treatment of the patients that followed the GOLD guideline included nebulized bronchodilators such as inhaled short-acting beta-2 agonists with or without short-acting bronchodilators, systemic corticosteroids, antibiotics when indicated, and oxygen supplement if necessary [5].

### 2.3. Outcome Measurements

The data collected included the patient demographics, ED triage levels as the Emergency Severity Index (ESI) version 4, [14] ED initial vital signs, history, physical examination, laboratory results, chest X-ray findings, treatment in ED before transfer to EDOU, ED length of stay, treatment given at EDOU, and disposition of the patients.

The primary variable was treatment failure of AECOPD patients at EDOU which was defined by the presence of at least one of the following: (1) death from respiratory cause during EDOU admission; (2) invasive or noninvasive mechanical ventilation; (3) EDOU length of stay >48 h or could not discharge from the EDOU within 48 h due to ward admission, intensive care unit (ICU) admission, or referral to another hospital; (4) COPD-related ED revisit within 72 h; (5) COPD-related readmission within 1 month; and (6) EDOU readmission within 1 month. Successful treatment of AECOPD patients was considered upon the absence of all these criteria.

### 2.4. Statistical Analysis

The statistical analysis was conducted using the R software version 3.6.1. Continuous variables were analyzed and are reported as mean ± standard deviation (SD) or median with the 1st and 3rd quartiles in the case of nonnormal distribution, while discrete variables are reported as absolute numbers and percentages. All data were based on nonparametric frequency distributions. The univariate model analyzed the baseline characteristics, clinical presentation, physical examination, management at the ED, and ED length of stay. The data were compared in subjects with treatment failure and treatment success. The Wilcoxon rank-sum test was used for continuous and ordinal variables, and Pearson's chi-squared test was used for categorical variables. Significant factors associated with treatment failure (*P* < 0.2) identified during univariate analysis were introduced into a logistic regression model with the backward stepwise selection scheme using Akaike's information criteria. First-order interaction terms with combinations of all independent predictors were introduced into the multivariate model one at a time. Generally, interaction terms were considered with statistical significance set at *P* < 0.05 and no significant interaction between the included variables in the final logistic regression models. Modeling results are shown as adjusted odds ratio (OR) with 95% confidence interval (CI). A *P* value <0.05 was considered statistically significant.

### 2.5. Compliance with Ethical Requirements

The Ethics Committee of the Prince of Songkla University approved this study. The Institutional Review Board of the Prince of Songkla University is affiliated with the International Conference on Harmonization in Good Clinical Practice or ICH-GCP protocol. According to our institutional review board protocol for waiver of informed consent, the requirement for consent was waived because the participants had no more than minimal risk and the patients received standard treatment. All research information was kept as confidential data in an encrypted file with password and limited data access by only the researcher and the assistant. The ethical registration number was REC.62-241-20-4.

## 3. Results

**Figure 1:**
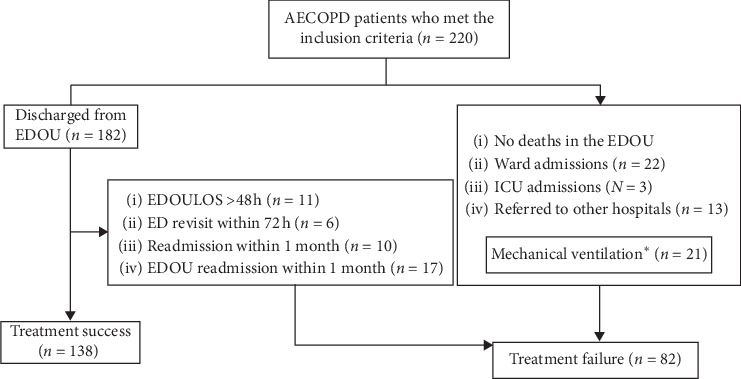
Study flow diagram. Abbreviations: AECOPD, acute exacerbation of chronic obstructive pulmonary disease; ED, emergency department; EDOU, emergency department observation unit; EDOULOS, emergency department observation unit length of stay; ICU, intensive care unit. ^*∗*^Patients who received either invasive or noninvasive mechanical ventilation or both and then needed ward admissions, ICU admission, or were referred to other hospitals.

A total of 220 patients met the inclusion criteria. Eighty two patients (37.3%) developed treatment failure. Twenty two patients (10%) were admitted to the general ward, three patients (1.3%) were admitted to the ICU, and 13 patients (6%) were referred to other facilities. Eleven patients (6%) were discharged from the EDOU with an EDOU length of stay >48 h. The numbers of patients who revisited the ED within 72 h, who were readmitted into the hospital within 1 month, and who were readmitted to the EDOU within 1 month were six (3.3%), 10 (5.5%), and 17 (9.3%), respectively. Twenty one patients (9.5%) received either invasive or noninvasive mechanical ventilation or both. No deaths occurred during the admissions to the EDOU (Figure 1).

The baseline characteristics that were not statistically significantly different in the treatment failure and treatment success groups were age, sex, smoking status, and GOLD classification. However, three baseline characteristics that had statistically significant differences were (1) history of admission for AECOPD in the previous year (*P*=0.026); (2) short-acting beta-agonist (SABA) use (*P*=0.001); and (3) underlying disease of diabetes mellitus (DM) (*P*=0.011) (Table 1). Characteristics of the study population at the ED related to the outcome of the COPD exacerbation that were not statistically significantly different were triage ESI, ED vital signs, history, physical examination, and laboratory investigation (Table 2).

Treatments of the two groups in the ED before transfer to the EDOU that were not statistically different and not related to treatment failure included total doses of bronchodilators (*P*=0.081), oxygen support (*P*=0.771), systemic corticosteroid therapy (*P*=0.713), and antibiotics (*P*=1.000). However, a shorter ED length of stay led to greater treatment failure (*P*=0.049) (Table 3).

Independent factors associated with treatment failure were introduced into a logistic regression model. The results are illustrated in Table 4. After adjusting for multiple factors, the study revealed that arrhythmias (odds ratio [OR] 3.8, 95% confidence interval [CI] 1.04–13.9), DM (OR 2.32, 95% CI 1.09–4.95), long-term oxygen therapy (OR 2.89, 95% CI 1.08–7.72), SABA use (OR 6.06, 95% CI 1.98–18.62), pneumonia findings on chest X-ray (OR 3.24, 95% CI 1.06–9.95), and ED length of stay <4 h (OR 2, 95% CI 1.08–3.73) were associated with treatment failure in the final model.

## 4. Discussion

This retrospective study demonstrated that the incidence of treatment failure in COPD patients with exacerbation admitted to the EDOU was 37.3%. The significant factors associated with treatment failure were arrhythmias and DM as comorbidities, long-term oxygen therapy, SABA use as baseline treatment, pneumonia findings on chest X-ray, and ED length of stay <4 h.

The large number of comorbidities in COPD patients significantly influenced the health care system that included health-related quality of life, resource consumption, and short-term prognosis [4, 5]. In this study, arrhythmias and DM were factors that increased the risk for treatment failure which were quite similar with previous studies [4, 11, 16, 17]. Sin et al. reported that cardiovascular disease including arrhythmias led to hospitalization of COPD patients, and arrhythmias was associated with higher in-hospital mortality [16]. García-Sanz et al. also reported that a history of atrial fibrillation was associated with 1-year and long-term mortality in COPD patients [18]. Tachyarrhythmia can worsen from sympathetic system activation when patients receive beta-2 agonists and anticholinergics or have hypoxic stress and complications which can influence mortality [18, 19].

DM also showed an increased risk of treatment failure due to poor glycemic control that reduces gas transfer due to microangiopathy [20]. Parappil et al. demonstrated that DM patients had increased length of stay and deaths compared with those without DM [17]. Furthermore, glucose levels greater than 11 mmol/L tend to reduce the immune response and depress neutrophil chemotaxis, phagocytosis, intracellular bacterial activity, opsonization, and cell-mediated immunity which impairs response to treatment in patients with AECOPD [17]. Furthermore, DM is associated with an increased risk for pulmonary infection and can worsen an AECOPD patient [21]. Finally, treatment of AECOPD with systemic corticosteroids may worsen the course of DM because it increases the risk of hyperglycaemia [22, 23]. AECOPD patients with comorbid DM require longer inpatient care in order to attain euglycemia prior to discharge [17].

A previous study reported an association between the use of long-term oxygen therapy at home and a higher risk of readmission and death in patients with AECOPD which was similar to this current study [1, 4, 11, 24, 25]. Patients with COPD who require constant oxygen therapy at home have limited movement and exercise ability which might affect morbidity and mortality [12]. Furthermore, COPD patients needing supplemental oxygen therapy suffer from severe hypoxemia and end-stage COPD. Therefore, it is not surprising to find an association between long-term oxygen therapy and higher mortality [24, 25].

This study showed that SABA use as the baseline treatment was related with treatment failure in AECOPD patients. This result was similar to a study by García-Sanz et al. which demonstrated that anticholinergic therapy as baseline treatment was associated with a higher incidence of serious adverse events in patients with AECOPD [11]. Also, Garcia-Aymerich said taking anticholinergics was related to an increased risk of readmission [12]. Since SABA and anticholinergic therapy have increased risk of tachyarrhythmia, it can be expected that both are related to higher hospitalization and in-hospital mortality.

Studies by Steer et al. and Shin et al. reported that AECOPD patients with pneumonia had a higher hospital mortality rate and mechanical ventilator support than those without pneumonia [26, 27]. Those results were consistent with our study which found that pneumonia in AECOPD patients was associated with increased treatment failure. But, it was contrary to a study by Boixeda et al. that reported no differences in the length of hospital stay, admission to the ICU, the need for mechanical ventilation, or in-hospital mortality [28]. Our data did not show severity of pneumonia which may have caused different results.

Surprisingly, an ED length of stay <4 h was related to a higher rate of treatment failure. This was possibly due to the longer time in the ED to complete evaluating and monitoring the patients to confirm they are safe for admission to the EDOU.

The strength of this study is the definition of treatment failure. We included all possible adverse outcomes for the patients. These were treated separately in many previous studies. The aim is to create the most appropriate criteria for admission of AECOPD patients to the EDOU. The limitations of this study are the small sample size and a small treatment failure group which possibly caused the study to be underpowered. Also, the data came from a single hospital and the results should not be generalized to other hospitals. Furthermore, other variable factors that may seem relevant to treatment failure, such as blood eosinophils, serum sodium, FEV1 <30% predicted, and treatment at discharge, were not obtained in this study [6, 29–31].

## 5. Conclusion

Several factors that were associated with treatment failure of AECOPD were identified in the current study which showed that 37.3% of AECOPD patients admitted to our EDOU developed treatment failure. Arrhythmias, DM, long-term oxygen therapy, SABA use, pneumonia findings on chest X-ray, and ED length of stay <4 h were important factors that affected treatment failure. Awareness of these factors may be beneficial in the design of future AECOPD patient dispositions in order to reduce treatment failure and improve the quality of management in the patients.

### 5.1. What Is Already Known on This Topic?

It is challenging to treat COPD patients with acute exacerbation in an emergency department observation unit. Recently, many studies have reported factors associated with treatment failure or adverse outcomes during hospital admission, but only a few studies focused on the emergency department observation unit. Moreover, there are only a few studies on the Thai population.

### 5.2. What This Study Adds?

This study showed other factors that are associated with treatment failure in patients with acute exacerbation of COPD in the emergency department observation unit.

## Figures and Tables

**Table 1:** Baseline characteristics of the study population.

Parameters	Treatment failure group (*n* = 82)	Treatment success group (*n* = 138)	*P* value
Age, years, median (IQR)	78.5 (70, 83)	76 (69, 82)	0.227
Male	74 (90.2)	124 (89.9)	1.000
Smoking status			0.631
Active smoker	8 (9.8)	20 (14.5)	
Ex-smoker	65 (79.3)	102 (73.9)	
Nonsmoker	7 (8.5)	10 (7.2)	
GOLD classification			0.506
Stage I	13 (15.9)	30 (21.7)	
Stage II	46 (56.1)	76 (55.1)	
Stage III	22 (26.8)	28 (20.3)	
Stage IV	1 (1.2)	4 (2.9)	
AECOPD in the previous year	71 (86.6)	108 (78.3)	0.176
Admission for AECOPD in the previous year	36 (43.9)	39 (28.3)	0.026
Intubation for AECOPD in the last 5 years	26 (31.7)	28 (20.3)	0.082
Baseline treatment			
Long-term oxygen therapy	13 (15.9)	10 (7.2)	0.074
Inhaled anticholinergic	74 (90.2)	126 (90.9)	0.982
Short-acting beta-agonist	78 (95.1)	107 (77.5)	0.001
Long-acting beta-agonist	76 (92.7)	121 (87.7)	0.345
Inhaled corticosteroids	76 (92.7)	118 (85.5)	0.168
Theophylline	52 (63.4)	80 (58)	0.513
Continuous oral antibiotics	8 (9.8)	5 (3.6)	0.078
Most frequent comorbidities			
Hypertension	46 (56.1)	62 (44.9)	0.143
Diabetic mellitus	22 (26.8)	17 (12.3)	0.011
Ischemic heart disease	8 (9.8)	12 (8.7)	0.982
Arrhythmias	8 (9.8)	5 (3.6)	0.078

*Notes*. Data are presented as *n* (%) unless indicated otherwise. Values <0.05 are statistically significant. Abbreviations: IQR, interquartile range; AECOPD, acute exacerbation of chronic obstructive pulmonary disease.

**Table 2:** Characteristics of the studied population in the ED related to the outcome of COPD exacerbation.

Parameters	Treatment failure group (*n* = 82)	Treatment success group (*n* = 138)	*P* value
ESI			1.000
ESI 2	76 (92.7)	127 (92)	
ESI 3	6 (7.3)	11 (8)	
Initial vital signs at ED			
Respiratory rate (breaths/min)	32.0 ± 5.3	31.0 ± 6.0	0.200
Systolic blood pressure (mmHg)	150.9 ± 24.9	149.3 ± 24.2	0.644
Diastolic blood pressure (mmHg)	81 ± 13.8	83 ± 16.1	0.356
Temperature (°C)	36.8 ± 0.7	36.8 ± 0.8	0.738
Pulse rate (beats/min)	96.3 ± 19.4	95.7 ± 18.6	0.805
SpO_2_ (room air) (%)	93.5 ± 4.4	94.4 ± 4.0	0.140
History			
Cough	72 (87.8)	118 (85.5)	0.782
Increased sputum/change color	27 (32.9)	49 (35.5)	0.808
Fever	21 (25.6)	34 (24.6)	1.000
Physical examination			
Accessory muscle use	20 (24.4)	30 (21.7)	0.774
Arterial blood gas			
pH	7.42 ± 0.04	7.42 ± 0.05	0.646
PaO_2_ (mmHg)	78.2 ± 33.6	73.1 ± 14.5	0.246
PaCO_2_ (mmHg)	33.4 ± 6.3	34.6 ± 7.0	0.305
SO_2_ (%)	93.9 ± 5.4	94 ± 4.8	0.920
PaO_2_/FiO_2_ (mmHg)	345.1 ± 83.6	345.3 ± 70.8	0.986
Lactate (mmol/L)	1.9 ± 1.0	1.9 ± 1.3	0.786
Most frequent abnormal CXR findings			
Pneumonia	9 (11)	7 (5.1)	0.173
Cardiomegaly	5 (6.1)	8 (5.8)	1.000
Bronchiectasis	0 (0)	4 (2.9)	0.299

*Notes*. Data are presented as mean ± SD or *n* (%) as appropriate. Values <0.05 are statistically significant. Abbreviations: ED, emergency department; ESI, Emergency Severity Index; CXR, Chest X-ray.

**Table 3:** Treatment of the studied population in the ED before transfer to the EDOU.

Parameters	Treatment failure group (*n* = 82)	Treatment success group (*n* = 138)	*P* value
Total doses of bronchodilator (nebulizer)	4.0 ± 1.4	4.4 ± 1.5	0.081
Oxygen support	18 (22)	34 (24.6)	0.772
Systemic corticosteroid	80 (97.6)	132 (95.7)	0.713
Antibiotic	39 (47.6)	66 (47.8)	1.000
Vital signs at ED before transfer to EDOU			
Pulse rate (beat/min)	99.7 ± 16.3	97.7 ± 13.7	0.344
Respiratory rate (breath/min)	25.9 ± 3.1	25.6 ± 3.6	0.525
Systolic blood pressure (mmHg)	134.6 ± 18.1	136.6 ± 20.4	0.457
Diastolic blood pressure (mmHg)	74.0 ± 11.8	74.2 ± 11.6	0.886
SpO_2_ (room air) (%)	95.5 ± 2.3	95.6 ± 2.2	0.726
ED length of stay (h), mean (SD)	3.8 ± 1.4	4.2 ± 1.6	0.049

*Notes*. Data are presented as mean ± SD or *n* (%) as appropriate. Values <0.05 are statistically significant. Abbreviations: ED, emergency department; EDOU, emergency department observation unit; SpO_2,_ oxygen saturation.

**Table 4:** Multivariable regression analysis of factors associated with treatment failure.

Factors	Unadjusted OR (95% CI)	Adjusted OR (95% CI)	*P* value
Arrhythmias	2.88 (0.91–9.11)	3.8 (1.04–13.9)	0.037
Diabetic mellitus	2.61 (1.29–5.28)	2.32 (1.09–4.95)	0.029
Long-term oxygen therapy	2.41 (1.01–5.78)	2.89 (1.08–7.72)	0.032
Short-acting beta-agonist use	5.65 (1.92–16.66)	6.06 (1.98–18.62)	<0.001
Pneumonia findings on chest X-ray	2.31 (0.83–6.45)	3.24 (1.06–9.95)	0.038
ED length of stay <4 h^*∗*^	1.93 (1.1–3.39)	2 (1.08–3.73)	0.026

*Notes*. Values <0.05 are statistically significant. Abbreviations: OR, odds ratio; CI, confidence interval; ED, emergency department. ^*∗*^According to the National Health Service (NHS) of the United Kingdom, a maximum length of ED stay should be 4 h to improve the quality of ED care [15]. The multivariate analysis of this study used a cut-point of ED length of stay at ≤4 h according to the NHS.

## Data Availability

All data are available within the article.
